# A genome-wide approach to link genotype to clinical outcome by utilizing next generation sequencing and gene chip data of 6,697 breast cancer patients

**DOI:** 10.1186/s13073-015-0228-1

**Published:** 2015-10-16

**Authors:** Lőrinc Pongor, Máté Kormos, Christos Hatzis, Lajos Pusztai, András Szabó, Balázs Győrffy

**Affiliations:** MTA TTK Lendület Cancer Biomarker Research Group, Research Centre for Natural Sciences, Magyar tudósok körútja 2, Budapest, H-1117 Hungary; 2nd Department of Pediatrics, Semmelweis University, Budapest, Hungary; Yale Comprehensive Cancer Center, Yale School of Medicine, New Haven, CT USA; MTA-SE Pediatrics and Nephrology Research Group, Budapest, Hungary

## Abstract

**Background:**

The use of somatic mutations for predicting clinical outcome is difficult because a mutation can indirectly influence the function of many genes, and also because clinical follow-up is sparse in the relatively young next generation sequencing (NGS) databanks. Here we approach this problem by linking sequence databanks to well annotated gene-chip datasets, using a multigene transcriptomic fingerprint as a link between gene mutations and gene expression in breast cancer patients.

**Methods:**

The database consists of 763 NGS samples containing mutational status for 22,938 genes and RNA-seq data for 10,987 genes. The gene chip database contains 5,934 patients with 10,987 genes plus clinical characteristics. For the prediction, mutations present in a sample are first translated into a ‘transcriptomic fingerprint’ by running ROC analysis on mutation and RNA-seq data. Then correlation to survival is assessed by computing Cox regression for both up- and downregulated signatures.

**Results:**

According to this approach, the top driver oncogenes having a mutation prevalence over 5 % included AKT1, TRANK1, TRAPPC10, RPGR, COL6A2, RAPGEF4, ATG2B, CNTRL, NAA38, OSBPL10, POTEF, SCLT1, SUN1, VWDE, MTUS2, and PIK3CA, and the top tumor suppressor genes included PHEX, TP53, GGA3, RGS22, PXDNL, ARFGEF1, BRCA2, CHD8, GCC2, and ARMC4. The system was validated by computing correlation between RNA-seq and microarray data (r^2^ = 0.73, *P* < 1E-16). Cross-validation using 20 genes with a prevalence of approximately 5 % confirmed analysis reproducibility.

**Conclusions:**

We established a pipeline enabling rapid clinical validation of a discovered mutation in a large breast cancer cohort. An online interface is available for evaluating any human gene mutation or combinations of maximum three such genes (http://www.g-2-o.com).

**Electronic supplementary material:**

The online version of this article (doi:10.1186/s13073-015-0228-1) contains supplementary material, which is available to authorized users.

## Background

Tumor evolution involves the accumulation of mutations during tumorigenesis enabling acquisition of the well-known hallmarks of cancer [1]. Current anticancer therapy presupposes a direct connection between mutational status and phenotype, therefore clinical decisions are often directly based on the genotype. For instance, KRAS status is considered an indicator of a patient’s response to the EGFR inhibitor panitumumab [2] and BRAF V600E status is used to predict response to the BRAF inhibitor vemurafenib [3]. However, such direct associations are not consistent among different clinical cohorts. For instance, vemurafenib was effective in BRAF V600E mutant melanoma but had no activity in BRAF V600E mutant colon cancer [4]. Similarly, mTOR and PI3K inhibitors have the same activity in PI3K mutant and normal breast tumors [5]. Also, the recently approved CDK4/6 inhibitor palbociclib had the same level of activity in breast tumors harboring altered and normal CDK4/6 [6].

The first NGS studies of breast cancer (BC) identified only three genes mutated in more than 10 % of BC tumors: TP53 (mutation rate 40 % of tumors), PI3K (25 % of tumors), and GATA3 (10 % of tumors). In particular, the most important genes identified in 100 primary breast cancers included AKT1, BRCA1, CDH1, GATA3, PIK3CA, PTEN, RB, TP53, ARID1B, CASP8, and MAP3K1 [7]. Banerji and coworkers studied 103 BC patients with NGS and recognized AKT1, PIK3CA, GATA3, TP53, and MAP3K1 as the most significant genes [8]. In the TCGA project, the most prevalent mutations hit PIK3CA, PTEN, AKT1, TP53, GATA3, CDH1, RB1, MLL3, MAP3K1, and CDKN1B [9]. Ellis and associates performed NGS on biopsies from two neoadjuvant aromatase inhibitor clinical trials and found PIK3CA, TP53, GATA3, CDH1, RB1, MLL3, MAP3K1, and CDKN1B to be the primary genes affected [10].

Despite differences in study design, these studies have a few common messages. First of all, NGS for a few hundreds of breast tumors has revealed new cancer genes. Additionally, NGS allowed the survey of intratumoral heterogeneity and has shown that there was always a dominant clone comprising at least 50 % of the tumor cells [11]. Therapeutically targeting the driver genetic aberrations will have the most significant effect on this clone. Targeted NGS in primary and metastatic breast cancers revealed that almost 85 % of tumors have a genetic aberration in a gene which is already actionable [12]. We must, however, note that some of the most important genes including TP53 and KRAS are not yet directly targetable.

The relationship between somatic mutations and clinical outcome is multi-layered, complex, and to a large extent unknown, therefore the assumption of a direct influence of genotype on phenotype is a key limitation in the generally accepted paradigm. An empirical analysis to associate sequence variations to outcome would need thousands of patients with sufficient follow up to be sequenced with next generation sequencing (NGS) technologies. We are far from this desirable goal. For instance, among the patients included in the Cancer Genome Atlas (TCGA) [13] – the largest breast cancer NGS database available today – approximately 89 % of the patients are censored. While one can expect fewer events because TCGA reports overall survival only, but this is coupled with a median follow up of merely 1.3 years.^1^ Thus, the paucity of clinical annotation of the TCGA and other mutation datasets makes direct testing of the prognostic impact both limited and underpowered especially if one is interested in survival differences in selected sub-cohorts of patients.

**Fig. 1:**
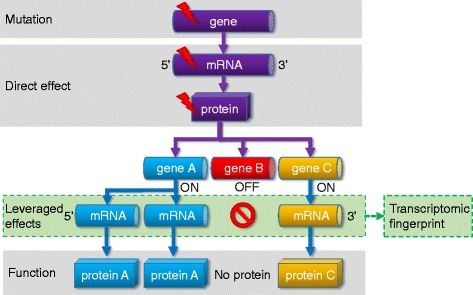
Complex effects of a single mutation. A cornerstone of our model is the leveraged effect of gene regulation network influencing the final consequence of a mutation. Some target genes will be suppressed while others will be amplified resulting in a markedly changed transcriptomic fingerprint for important genes

Here we propose an approach that could circumvent this limitation. We postulate that a somatic mutation perturbs not only the mutated gene but the perturbation will propagate to a network of functionally related genes from the same or other cellular pathways. As a result, the effect of the mutation will be leveraged by a set of genes, and the changes in the expression of these genes that we term a ‘transcriptomic fingerprint’ can be used as a surrogate marker of the mutation status (Fig. 1).

Naturally, the genes indirectly affected by a mutation are unknown, but can be inferred by statistical means if there are sufficient data available. As we have recently shown, genome-wide transcriptomic analysis across all genes can identify genes affected by a given mutation [14]. In brief, a large number of patients with known genotype are divided into two cohorts (those with mutations in a given gene or wild type), and transcripts associated with mutational status are identified by ROC analysis across all genes. These genes can then be evaluated for correlation with clinical outcome including therapy response [14].

In the present work we extend this analysis to predict the probability of patient survival from mutational data of breast cancer patients. As already mentioned, there is a growing body of samples with both mutation and gene expression data, but only a few datasets include relapse events on the same patients. At the same time, microarray (gene chip) data have been collected that have substantially longer follow up times and extensive clinical annotations. Harvesting the combined prognostic value of these two kinds of data is a challenging task with a potential for exceptional clinical relevance. Here, our aim was to combine available genotype and RNA-seq data generated by NGS with gene expression data generated by gene chips, in order to develop a tool that could estimate the complex prognostic impact of a genomic anomaly. We believe that this method and the associated online tool can be used to prioritize genes for further functional study.

## Methods

**Fig. 2:**
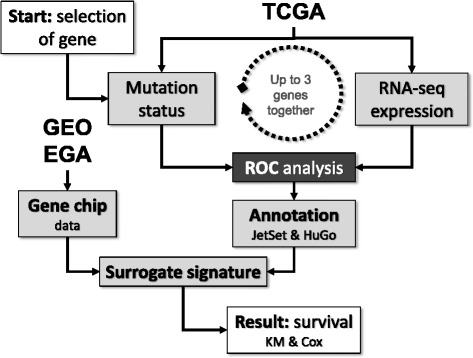
Overview of the analysis setup. Mutation status and gene expression levels obtained from the TCGA repository are compared using ROC analysis to identify a gene expression signature for each mutation. Then, the ability to predict survival for this signature is assessed in the gene chip dataset. The entire analysis can be made for a single gene or for up to three genes together

We implemented an approach to predict survival by simultaneously using gene mutation status and gene expression data. Main steps of the algorithm termed G-2-O (genotype to outcome) involve splitting TCGA samples into two cohorts using mutation status of the gene(s), then identifying differentially expressed genes between these cohorts using ROC analysis, and rendering a survival analysis using an independent dataset established using gene chip data. The entire statistical pipeline is summarized in Fig. 2 and the individual steps as well as database construction are described below. An online interface for the G2O algorithm is available at http://www.g-2-o.com.

### NGS data download

Whole exome sequencing data and RNA-seq data for breast cancer patients were obtained from The Cancer Genome Atlas (TCGA) of the National Cancer Institute (http://cancergenome.nih.gov/) [13]. The aligned TCGA datasets were downloaded from the CGHub repository (website: https://cghub.ucsc.edu/) using the CGHub download client software GeneTorrent (version 3.8.5) for both tumor samples and matched normal samples (total n = 1,526).

### Mutation calling

Mutation calling and annotation was done with *MuTect* [15] using default parameters. The human reference genomes GRCh37, GRCh37-lite, and HG19 used for mutation calling were downloaded according to the CGHub websites’ ‘Reference Assemblies’ guideline (available at https://browser.cghub.ucsc.edu/help/assemblies). To reduce the total number of mutations, we only accepted somatic mutations that were labeled as ‘KEEP’ according to the *MutTect* judgment algorithm, and were present in at least four reads with a minimum of 20-fold read coverage. Our rationale behind the selection of a lower stringency threshold was intra-tumor heterogeneity, that is, the presence of multiple, genetically diverse clones in a single tumor which may affect tumor growth and patients survival. During treatment minor clones can also have a prominent effect on the patients’ response to therapy and survival [16]. Finally, the identified mutations were annotated with *MuTect* using the dbSNP (build 139) and COSMIC (version 68) databases [17].

### Hit annotation

The identified sequence variations were functionally annotated using SNPeff v3.5 [18]. The reference databases used with SNPEff were downloaded with the SNPeff downloader. SNPeff is capable of annotating VCF files generated by MuTect. The annotation generated two outputs including a functionally annotated VCF file and a list containing all the genes and the effect the mutations had on the genes. The applied filters for the ‘Function class’ were: coding non-synonymous, stop gain, coding synonymous, locus region of gene, and splice site. The complete gene list comprising of n = 22,938 genes from SNPeff was used during the functional annotation (see Additional file 1: Table S1).

### Processing of copy number variations

Copy-number variation (CNV) data were downloaded from the TCGA repository on 1 February 2015. The CNV data were filtered according to two parameters: at least 10 probes had to be present at a position with a segment mean above 0.2 for amplification, and under −0.2 for deletions, as suggested in [19]. The filtered segments were annotated using the Human Gene Sets GTF annotation file downloaded from the Ensembl database of the Human Genome version GRCh37.

### Processing of RNA-seq data

RNA-seq data for breast cancer patients was obtained from the TCGA repository for the same patients who also had mutation data. We downloaded the pre-processed level 3 data generated using the Illumina HiSeq 2000 RNA Sequencing Version 2 platform. Expression levels for these samples were determined using a combination of MapSplice and RSEM. Individual patient files were merged into a single database using the plyr R package [20].

### Gene chip data

Set-up of microarray-database using GEO and EGA-available microarray datasets was established as described previously [21]. The entire breast cancer gene chip database contains 5,934 patients. The raw Affymetrix .CEL files were MAS5 normalized in the R v3.0.2 statistical environment (http://www.r-project.org) using the *Affy Bioconductor* library. We selected MAS5 for normalization because it performed among the top normalization methods compared to RT-PCR measurements in our previous project [22]. Array quality control was computed as described previously [23]. For each gene, the most reliable probe set was selected using *JetSet* [24].

### Validation dataset

As a starting point of our analysis, we wanted to confirm the correlation between the Affymetrix microarray and Illumina RNA-seq platforms. For this purpose, we used the TCGA lung squamous cell carcinoma dataset (LUSC), because it contains matched RNA-seq and Affymetrix gene chip data on the same samples. A matched gene list comprising 10,987 genes was prepared and pre-processed as described above for the breast cancer datasets. For each gene, we used the Spearman rank correlation to compare RNA-seq and gene-chip based expression levels. The correlation and a *P* value were calculated R using the *rcorr* function of the *Hmisc* package (http://biostat.mc.vanderbilt.edu/wiki/Main/Hmisc).

### G-2-O algorithm

The first step of the algorithm involves assigning each sample to one of two cohorts based on the mutation status of the investigated gene. Generally, the type of DNA mutation used to split the samples can be any alteration in the gene. As an output of this step a binary vector termed ‘mutation pattern’ is calculated. In this, affected samples are represented by a ‘1’ and unaffected samples are represented as ‘0’.

In certain settings one might be interested in a custom selection of gene variations – we performed such an analysis to compare mutations resulting in altered mRNA sequence (including start gain, exon, splice site, non-synonymous coding, stop gain, and stop loss mutations) to silent mutations (including mutations in introns and synonymous coding mutations). In the final analysis interface we enabled the user to custom-select a combination of the alterations.

The next step is categorizing genes whose expression is different between the two cohorts designated in the previous step. For this, a univariate receiver operating characteristic (ROC) analysis is separately performed using the expression values of each gene. In other words, samples are compared based by the previously identified ‘mutation pattern’ and significantly altered genes are selected based on the area under the curve (AUC) value, and the associated *P* value computed in the ROC analysis. The AUC value is calculated by the ROCR package using the *prediction()* and *performance()* functions. The G2O algorithm calculates the *P* values where the null hypothesis is that the AUC value equals to 0.5. First, the script calculates the standard error of the null hypothesis [25] from which the function derives the z-score. The *P* value is then calculated by transformation of the z-score using the normal distribution. Only genes passing both AUC and *P* value thresholds are considered significant. The final output of this step is a list of up- and downregulated genes whose expression is significantly associated with the given genotype alteration of the original input gene.

The final step involves the calculation of survival in the gene chip database using the ‘metagenes’ selected in the TCGA data – the average expression of the set of significant genes identified by the ROC analysis are designated as the ‘metagene’ for the given genotype. This survival analysis is therefore independent from the genotype and RNA-seq data.

We then assessed the correlation with survival for each metagene using Cox proportional hazards regression and by plotting Kaplan-Meier survival plots for median-dichotomized metagenes. In each analysis, metagenes for up- and downregulated gene sets are treated separately resulting in two survival analyses for each genotype (up- and downregulated genes in tumors having a mutation of the gene).

### Statistical packages utilized in the G-2-O algorithm

The ROC analysis is performed using the ROCR [26] Bioconductor (http://www.bioconductor.org/) library in the R statistical environment (http://www.r-project.org). The AUC values are calculated automatically by the ROCR package using the *prediction()* and *performance()* functions. By default, a significance threshold of 0.01 and a minimal AUC of 0.65 are required for each gene to be considered as significant. Cox regression analysis is executed with the ‘survival’ R package version 2.38 downloaded from CRAN (http://CRAN.R-project.org/package=survival). The Kaplan-Meier plots are generated using the ‘survplot’ R package developed by Aron Eklund (http://www.cbs.dtu.dk/~eklund/survplot/). Threshold for statistical significance in the survival analysis was set at *P* <0.05 and average HR >1.4 (average HR is based on the mean of HR in the cohort having a higher HR and 1/HR of the cohort having a lower HR value). In the entire analysis pathway, none of the samples involved in the training (ROC analysis) are included in the test (survival analysis) as well.

### Permutation test and random holdout

To assess the robustness of the results, we performed the entire analysis 100× with 100 randomly selected genes in each run and counted the number of significant results. To assess the false positive rate, we plotted the number of significant genes against the number of runs delivering the given number of significant genes.

In a second cross-validation analysis set up to estimate the reproducibility of the results, we excluded 20 % of the samples at random and re-ran the entire analysis 10 times for a set of 20 selected top driver candidate genes. We derived the average *P* value as well as the standard deviation of all analyses for the given gene. Up- and downregulated gene sets were treated separately in this analysis.

## Results

### Database setup

Central to our approach is the joint analysis of three breast cancer datasets, including somatic mutations and RNA-seq gene expression from the TCGA project and microarray and detailed survival data for a separate large cohort of breast cancer patients. Mutations were identified in 20,938 genes in 763 patients. RNA-seq expression data for 10,987 genes was also available for the same tumors - only genes also present in the gene chips were utilized to facilitate translation between the two platforms. The microarray data for the matched 10,987 genes, and detailed follow-up including survival were available for 5,934 patients from 39 independent breast cancer datasets.

### Comparison of RNA-seq and gene chip data

A total of 129 LUSC patients had matched RNA-seq and microarray data. In these, Spearman correlation was computed across all genes within each patient separately, the median correlation was 0.73 with a *P* value <1E-16. The coefficient was higher than 0.68 in all cases, indicating a robust correlation. Thus, we concluded that the planned utilization of gene chips as a surrogate of RNA-seq data is a feasible strategy for our analysis.

### Analysis interface

**Fig. 3:**
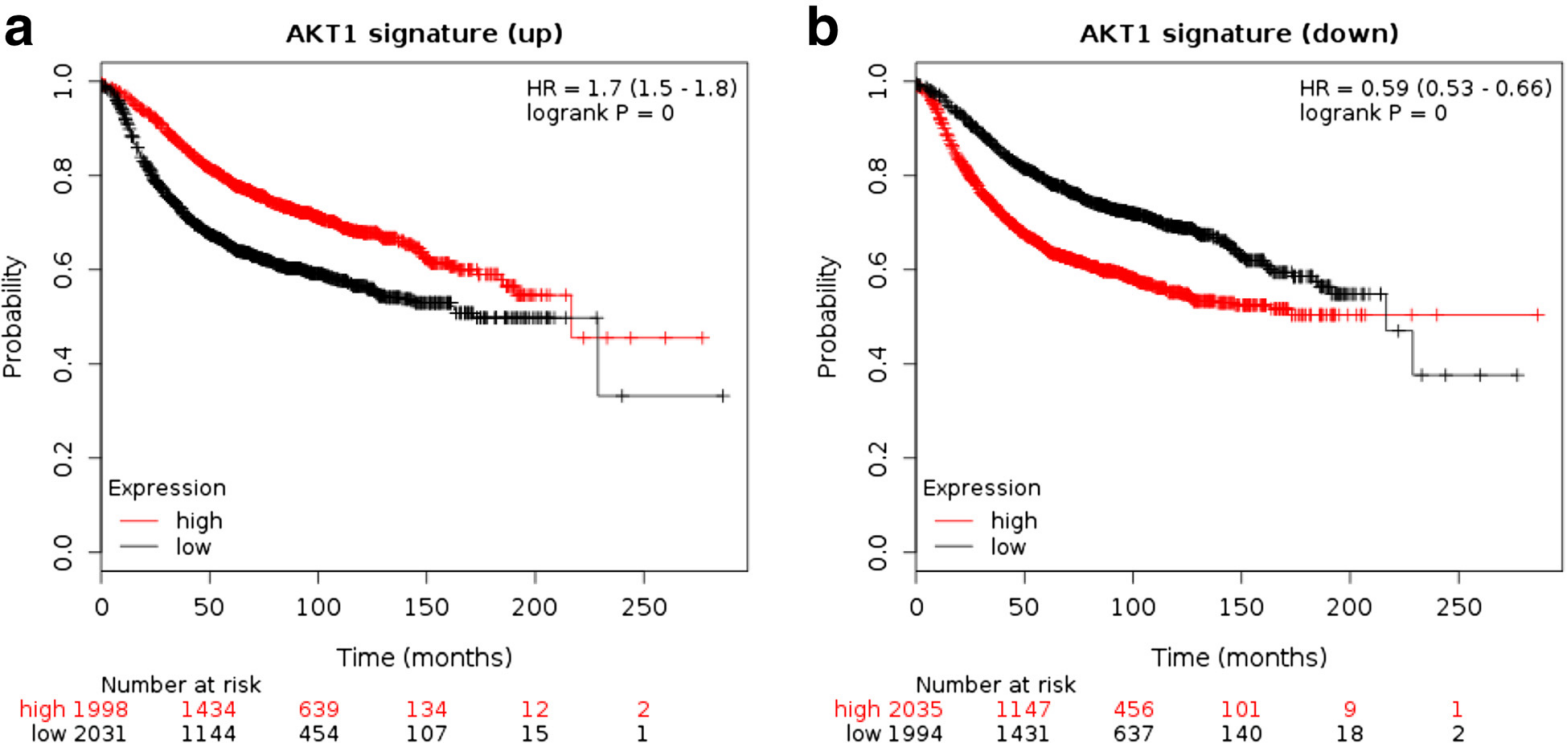
A sample analysis result for the AKT1 gene. The plots show the effect of both upregulated genes (**a**) and downregulated genes (**b**). Notice the robust inverse correlation: higher expression of upregulated genes results in worse relapse free survival while lower expression of downregulated genes also leads to worse survival. High and low: compared to the median of the surrogate gene expression signature

We have set up an integrated web server that takes a few user-selected genes as input. Then, it: (1) looks up somatic mutations in the gene(s); (2) computes the combined transcriptional fingerprint of the mutation(s) using ROC analysis of breast cancer RNAseq data; and (3) uses the top up and down metagenes to estimate patient survival using Cox regression on gene chip data. An important element is estimation of the transcriptional signature for each somatic mutation, which is carried out by ROC analysis on the mutation and RNA-seq data. The output includes Kaplan-Meier plots for both up-and downregulated signatures. As an example, see Fig. 3 for the gene RFC1.

### Mutation landscape of the training database

We compared the output generated by our mutation detection method derived from the raw exome sequencing data to previously reported common mutations in breast cancer. Here we omitted any post-processing to truly assess the established pipeline without the influence of confounding additional steps. The computed mutation prevalence for the known cancer genes overlapped with those of previous studies. We listed the top 20 cancer-related and non-cancer-related genes in Table 1. Genes listed in the COSMIC database [17] were designated as cancer-related.

**Table 1:** A list of the top 20 most common cancer and non-cancer (based on Cosmic) mutations identified by analyzing 763 breast cancer samples

Cosmic genes^a^		Non-cosmic genes	
Gene	% of samples w/ mutations	Gene	% of samples w/ mutations
FRG1B	58.1	NBPF1	78.2
TTC34	36.5	BAGE2	70.6
PIK3CA	34.6	KMT2C	37.9
CROCC	32.9	BAGE5	34.7
RYR2	30.8	TTN	33.6
TP53	28.1	CROCCP2	33.6
HYDIN	28	MUC16	28.4
NBPF14	24.9	ROCK1P1	23.7
CSF2RA	23.9	TPTE2P6	18.5
PTPRN2	23.9	MST1L	15.9
EIF2B5	23.8	HERC2P4	14.3
PCDHGA1	23.4	MST1P2	14.0
SYNE1	23	DDX12P	13.6
SPDYE3	22.9	DPY19L2P1	12.8
HMCN1	21.2	ANKRD20A9P	11.5
DDX11	20.9	MLLT10P1	11.1
GON4L	20.8	TBC1D3P1-DHX40P1	11.1
PKHD1L1	20.8	MROH7-TTC4	11.0
NEB	20.3	AQP7P1	10.8
USH2A	20.2	SDHAP1	10.6

^a^Version: COSMICv71

A significance threshold of 0.01 and a minimal AUC of 0.65 are required for each gene to be considered as significant in the ROC analysis, and the threshold for significance in the survival analysis was set at *P* <0.05 and average HR >1.4. This analysis included all genes (including the established driver genes as well)

### Comparison of mutation calling results to the published TCGA data

Since our method using MuTect identified more mutations than the available mutations downloaded from the publicly available TCGA Data Matrix repository (https://tcga-data.nci.nih.gov/tcga/dataAccessMatrix.htm), we performed a mutation comparison between our results and the downloaded TCGA MAF files. Our mutation calling and annotating pipeline identified a significantly higher number of mutations (mean ± standard deviation of identified alterations by MuTect: 644 ± 69/sample and TCGA: 102 ± 14/sample), which spawn from (1) lower coverage threshold; (2) lower mutation frequency threshold; (3) mutation annotation with snpEff generated multi-gene annotations; and (4) we accepted mutations that were found either in intronic or upstream regions of a gene.

While the TCGA processing using a consensus call of three different centers was set up to achieve high true positive rate, the proportion of false negatives may also be high. On the contrary, while MuTect identified more somatic mutations, only 16 % of these fall into gene coding regions (usually with lower coverage and/or mutation frequencies). The majority (84 %) of mutations identified were either found in introns, or upstream regions of a gene. When examining the 10 genes with the highest prevalence in the TCGA MAF files, the differences in prevalence compared to the MuTect mutation calling were minimal (see Additional file 2: Table S2). To validate MuTect results, we visualized a few examples in the IGV Browser [27] (see Additional file 3: Figure S1a–f). When evaluating the entire set of all single nucleotide variations across all 763 samples, our analysis pipeline identified 1,636 of the 1,752 alterations published in the TCGA repository, which translates to an intersection of 93 %.

### Identification of strongest driver genes

To focus on genes with high clinical relevance, we aimed to identify the strongest driver gene candidates. These analyses were run by accepting an AUC value over 0.65 and a *P* value below 0.01 as significant in the ROC analysis and accepting a HR over 1.4 and a *P* value below 0.01 in the survival analysis. The complete analysis results for both up- and downregulated genes sets for each of these 176 genes are listed in Additional file 4: Table S3 and the 20 best performing genes based on the computed HR are listed in Table 2. The complete set of all genes included in the metagenes for each of these drivers is listed in Additional file 2: Table S2.

**Table 2:** Analysis results for the top genes including new and already established driver gene candidates. The table is split according to top 10 oncogene candidates (A) and top 10 tumor suppressor gene candidates (B)

Gene	% of samples w/ mutations	Genes from ROC analysis (n)	Upregulated genes		Downregulated genes	
			*P* value	HR	*P* value	HR
(A)						
AKT1	5.5	136	<1E-16	1.8	1.60E-15	0.64
ATG2B	5.1	20	6.70E-09	1.4	6.90E-14	0.66
COL6A2	5.3	151	3.60E-13	1.5	1.50E-09	0.72
MTUS2	5.8	97	6.90E-06	1.3	1.10E-16	0.63
OSBPL10	5.2	46	2.40E-08	1.4	1.80E-12	0.68
POTEF	5.7	16	1.60E-11	1.4	5.60E-16	0.64
SCLT1	5.5	27	3.90E-08	1.4	5.10E-13	0.67
TNC	6	15	1.10E-07	1.3	<1E-16	0.63
TRANK1	5.3	10	<1E-16	1.7	1.90E-05	0.79
TRAPPC10	6.1	3	<1E-16	1.7	2.60E-11	0.69
(B)						
ARFGEF1	6.4	63	<1E-16	0.52	<1E-16	1.7
BRCA2	6	74	<1E-16	0.52	2.20E-16	1.6
GGA3	5.5	359	<1E-16	0.49	1.40E-14	1.5
MPP6	5.5	48	<1E-16	0.53	<1E-16	1.8
PHEX	6.6	20	<1E-16	0.47	2.60E-15	1.5
PXDNL	6.1	255	<1E-16	0.51	<1E-16	1.6
RGS22	6	350	<1E-16	0.5	<1E-16	1.7
TP53	28.3	1566	<1E-16	0.48	5.60E-16	1.6
UBR5	9.9	8	<1E-16	0.53	<1E-16	1.7
UNC5D	6.6	51	<1E-16	0.54	<1E-16	1.8

Significant signatures identified had to have an AUC value over 0.65 and a *P* value below 0.01 in the ROC analysis and an average HR over 1.4 and a *P* value below 0.01 in the survival analysis

HR and *P* value: results of the Cox regression for both up- and downregulated genes

To compare our results to more stringent mutation calling criteria, we re-computed the entire analysis by using the mutation calls published by TCGA. Out of the 176 driver genes identified by the basic G-2-O algorithm 61 genes were found significant, 61 genes delivered ‘NA’ results, and 54 genes were not significant. The result was always ‘NA’ in cases when there were fewer than seven patients with a mutation in the given gene. Of the 61 significant genes, the correlation with survival was matching for 55 genes, an opposite correlation was observed for six genes. In other words, by introducing the TCGA thresholds, 65 % of the driver genes were not identified, mainly because of insufficient number of genotype alterations.

### Computation of expected positive rate

**Fig. 4:**
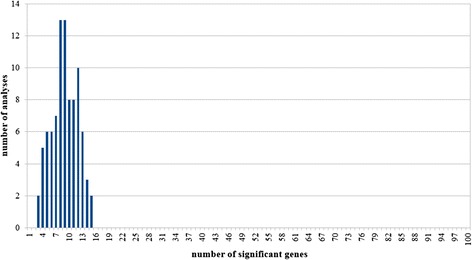
Distribution of significant hits in random analyses. To assess the expected false positive rate of the method, the entire pipeline was run 100 times each time on 100 randomly selected genes. The mean number of significant genes was 9.24, the number of significant genes was not more than 15 in any run

To estimate the expected false positive rate, we performed a random resampling test in which we repeated the analysis 100 times, each time running the pipeline on 100 random genes. The mean number of significant genes was 9.24, none of the runs delivered more than 15 significant genes, and there were at least three genes significant in each analysis. The number of runs having a given number of significant genes is depicted in Fig. 4. To estimate the number of false positive hits we compared the results (924 hit per 10 k genes) to the number of COSMIC cancer consensus genes (n = 571) using following formula: FPR = FP/(FP + TN), where FPR = false positive rate, FP = false positives, TN = true negatives. The estimated FPR was at 5 % on average (range 0–10 %).

### Assessing reproducibility by random holdout

In order to estimate the reproducibility of the results, we excluded 20 % of the samples at random and re-ran the entire analysis ten times for a selected set of high prevalence genes including 20 top driver candidate genes (AKT1, PIK3CA, TP53, BRCA2, and so on). Titin (TTN) was also selected as a non-cancer gene because it has a high mutation prevalence probably due to its massive size. Across all analyses, the AKT1 gene upregulated gene signature had an average hazard ratio of 1.7 (range 1.6–1.8) with an average *P* value of <1E-16 (<1E-16 – <1E-16), paired with a downregulated gene signature average hazard ratio of 0.72 (0.59–0.87) with an average *P* value of 2.5E-3 (<1E-16–1.4E-2). In the case of PIK3CA, the upregulated gene signature hazard ratio was 1.3 (1.2–1.6) with an average *P* value of 1.6E-4 (<1E-16–8.8E-4), paired with a downregulated gene signature hazard ratio of 0.64 (0.53–0.7) with an average *P* value of 7.2E-12 (<1E-16–4.3E-11). The remaining putative driver genes delivered similar outcomes, the complete results for all genes are listed in Additional file 5: Table S4. The TTN gene had no significant results in any of the analyses.

Finally, we set up the ROC analysis to enable utilization of different thresholds for accepting a gene as significant. These include an AUC value between 0.6 and 0.75 and a *P* value between 0.05 and 0.0001. To estimate the effect of different cutoffs on classification performance, we performed the analysis using each available cutoff (for example, for AUC and *P* value) combinations for TP53, PIK3CA, and EGFR. In these analyses, the resulting HR and *P* values showed less than 2 % deviations (data not shown).

## Discussion

We developed a genome-wide approach to link genotype to clinical outcome by consecutively utilizing next generation sequencing and gene chip data of 6,697 breast cancer patients. The two key elements of this system are: (1) the link between NGS datasets and the well-annotated gene-chip datasets; and (2) the assumption that the link is not one-to-one, but includes a set of genes (transcriptomic fingerprint) indirectly affected by a somatic mutation. We have set up a web-based system that enables utilization of the entire analysis pipeline to assess the correlation between mutational status and survival for 10,987 genes. Raw data for each of the available samples were downloaded and re-processed using the same analysis pipeline to enable robust utilization of the data. Genes correlated with a given genotype were identified by employing a ROC analysis across all genes. Simultaneous utilization of RNA-seq and gene chip assays to measure expression of the same genes is supported by the recent validation of high concordance between these technologies [28].

While microarray and RT-PCR based assays are limited to measuring the expression of selected, *a priori* chosen genomic features, next generation sequencing (NGS) technologies are not subject to this limitation and can be used to evaluate whole genomes, targeted genome regions, transcriptomes, and epigenomes. A primary goal of an NGS analysis is the identification of driver mutations – changes conferring selective advantages to cancer cells and thus contributing to tumor expansion. However, driver mutations are often buried inside a cloud of passenger mutations – changes without biological significance, most likely products of the genomic instability in cancer cells.

As of today, there are no validated drivers of metastatic disease and drivers of therapy resistance. In addition, genes imperative for therapy decision including HER2 (mutation in 1.5 % of samples) and ESR1 (mutation in 0.6 % of samples) show a very low mutational prevalence. These facts emphasize that many driver genes are not yet identified. Here, we provide an alternative approach to identify new driver genes. We hypothesize that by measuring the leveraged effect of a given mutation on the overall transcriptomic profile of the tumor the effects of that genomic change can be evaluated. The utilization of this approach on already established driver genes confirmed our method. We have to note that some genes tend to have high false discovery rate in mutation calling, for instance HYDIN, SYNE1, and USH2A [29] – it is an advantage of the proposed methodology is that despite the high mutation rate, none of these genes reached the significance necessary to be included in the list of putative driver genes.

A potential limitation of our method is the assumption that a direct link exists between mutation changes and gene expression. In fact, a mutation in a driver gene can be silent without changes in the transcriptional fingerprint – for example activation of certain signaling pathways like PIK3, which has been shown to be independent of changes in expression [30]. Another restriction of our approach is that it cannot be validated for genes with mutation in a few patients only (low mutation prevalence below 1 %). A further limitation is that due to lack of data we had to disregard epigenetic effects on transcription. The same mutation with or without a methylation event might result in different transcriptional consequences.

We have also computed the expected number of significant genes selected at random, which yielded an average of 9.2 significant genes per 100 randomly picked genes. This analysis includes a proportion of false positive hits in addition to the known driver genes (5 % of all genes based on COSMIC consensus genes). Nonetheless, an alternative explanation is that there are still a large number of unidentified driver genes. Altogether, the overall number of false positive results is far below the output of random multigene signatures – 90 % of which delivered significant association with survival in breast cancer in a previous study [31]. We have to note that these random signatures contained more than 100 genes and the G-2-O metagenes contain less than 100 genes in most of the analyses.

Direct analysis on the prognosis of a given mutation based on the TCGA data is possible using the cBioPortal [32]. However, shortcomings of the cBio portal include utilization of overall survival data only, short follow-up, few death events, and unbalanced cohorts dependent on prevalence of the mutation. Furthermore, today we know that breast cancer subtypes carry different sets of mutations and are linked with different baseline prognoses – these differences can be easily detected using the G-2-O analysis platform which enables one to investigate the effect on prognosis in each of the molecular subtypes separately. For example, the hazard ratio associated with a PIK3CA mutation in ER positive and negative tumors deliver completely different prognostic implications (for the upregulated gene signature is the HR in ER negative samples 0.66 and HR in ER positive tumors is 1.2).

Previously, an integrated algorithmic approach termed DriverNet was set up to analyze population-based genomic and transcriptomic interrogations of a tumor to identify pathogenic driver mutations [33]. Paradigm, another similar project was developed to identify driver pathways [34]. Both of these approaches were based on incorporating known associations or interactions between genes. In contrast, the G-2-O approach is fundamentally different as no recognized gene or pathway interaction data are included in the analysis. Rather, the association between genes is re-computed for each gene in each analysis – this approach delivers results not influenced by *a priori* association networks.

## Conclusions

To date, targeting of recognized driver genes delivered suboptimal results in breast cancer [35]. There are two major reasons behind this phenomenon – only a handful of genes are already actionable while most of these known driver genes have a prevalence below 10 % of patients. Thus, we need to identify clinically relevant mutations to spot candidate genes for targeted therapy or for new personalized clinical trials. Here, we have set up a pipeline enabling the functional validation of a discovered mutation for any gene in a large breast cancer cohort by computationally connecting genotype to an extended, surrogate gene expression signature and utilizing this signature in gene chip datasets. Gene chip databases already serve as transcriptomic basis for cross-dataset analysis tools like Breast-Mark [36] or the KM-plotter [37]. Since the gene expression datasets are large and reasonably well annotated, we can use the gene expression surrogate to test the prognostic significance of a DNA level change. The registration-free, online interface of the Genotype To Outcome (http://www.g-2-o.com) web server enables researchers, bioinformaticians, and clinicians to query the genetic background of a patient by performing an express evaluation of a discovered mutation, or combinations of mutations for any gene.

## Additional files

Additional file 1: Table S1. List of genes used during mutation annotation with SnpEff. (TXT 973 kb) Additional file 2: Table S2. The complete set of all genes included in the metagenes for each of the putative driver genes listed in Table 2. (XLSX 156 kb) Additional file 3: Figure S1. Somatic mutation found by MuTect and not present in the TCGA MAF file for the genes RNU5-F1 (A), SH3PXD2A (B), PSD3 (C), MYOC (D), CES3 (E), and GPR113 (F). (PPTX 378 kb) Additional file 4: Table S3. Analysis results for both up- and downregulated genes whose AUC value was over 0.65 and *P* value below 0.01 in the ROC analysis and accepting a HR over 1.4 and a *P* value below 0.01 in the survival analysis. (XLSX 18 kb) Additional file 5: Table S4. Complete results of the random holdout analysis. The table contains the average HR and *P* values computed in the survival analysis, as well as their minimum and maximum values. (XLS 27 kb)

## Footnotes

1 Overall survival event available for 87 patients out of 763, median follow-up 487 days. TCGA repository queried 23 February 2015.
