# Supplementary figures and images for: A genome-wide approach to link genotype to clinical outcome by utilizing next generation sequencing and gene chip data of 6,697 breast cancer patients
Source: Genome Med. 2015 Oct 16;7:104. doi: 10.1186/s13073-015-0228-1 (PMC4609150; doi:10.1186/s13073-015-0228-1)

## Slide 1
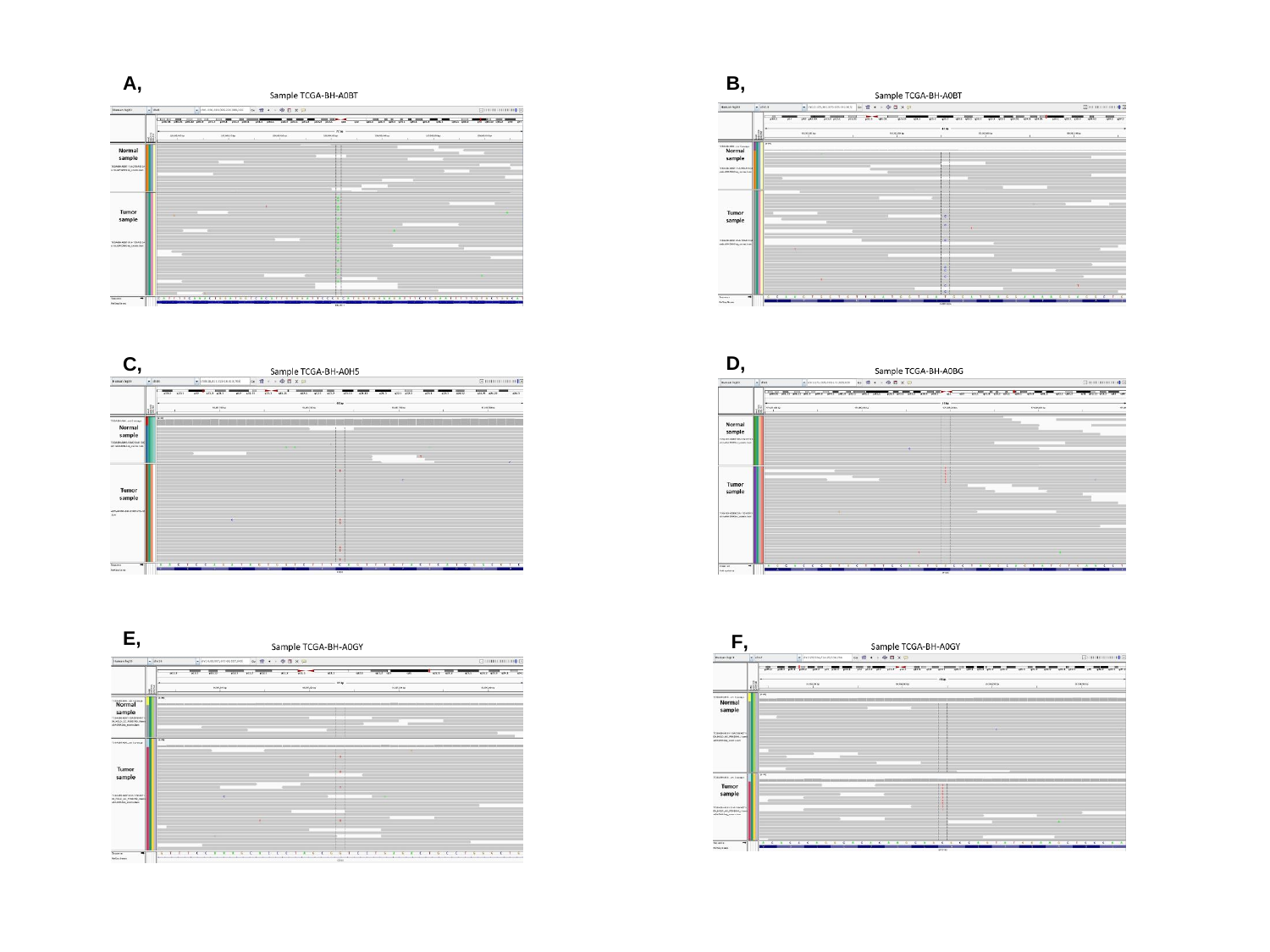

A,
B,
D,
C,
E,
F,

Supplement: Additional file 3: Figure S1. — Somatic mutation found by MuTect and not present in the TCGA MAF file for the genes RNU5-F1 (A), SH3PXD2A (B), PSD3 (C), MYOC (D), CES3 (E), and GPR113 (F). (PPTX 378 kb) [file 13073_2015_228_MOESM3_ESM.pptx]
